# Identification of polymorphisms associated with attenuation of Vif and Vpr in HIV-1 Elite Controllers

**DOI:** 10.1590/0074-02760240274

**Published:** 2025-06-27

**Authors:** Suwellen Sardinha Dias de Azevedo, Fernanda Heloise Côrtes, Mariza G Morgado, Brenda Hoagland, Larissa M Villela, Beatriz Grinsztejn, Valdilea Gonçalvez Veloso, Gonzalo Bello

**Affiliations:** 1 Fundação Oswaldo Cruz-Fiocruz, Instituto Oswaldo Cruz, Laboratório de AIDS & Imunologia Molecular, Rio de Janeiro, RJ, Brasil; 2 Fundação Oswaldo Cruz-Fiocruz, Instituto Nacional de Infectologia Evandro Chagas, Laboratório de Pesquisa Clínica em DST/AIDS, Rio de Janeiro, RJ, Brasil; 3 Fundação Oswaldo Cruz-Fiocruz, Instituto Oswaldo Cruz, Laboratório de Arbovírus e Vírus Hemorrágicos, Rio de Janeiro, RJ, Brasil

**Keywords:** HIV-1, elite controllers, accessory genes, attenuating mutations

## Abstract

**BACKGROUND:**

Elite controllers (ECs) are a rare subset of individuals who naturally suppress human immunodeficiency virus type 1 (HIV-1) replication in the absence of antiretroviral therapy. Specific polymorphisms in the accessory proteins Vif and Vpr have been associated with diminished viral fitness *in vitro* and are more frequently detected in ECs compared to other individuals infected with HIV-1.

**OBJECTIVE:**

To assess the frequency of gross genetic defects or polymorphisms that may attenuate the function of the HIV-1 accessory proteins Vif and Vpr within the proviral quasispecies of ECs.

**METHODS:**

We performed single-genome amplification (SGA) and sequence analysis of the proviral quasispecies of the accessory genes *vif* and *vpr* in samples obtained from eight ECs with over 10 years of suppressive viral control and no evidence of disease progression.

**FINDINGS:**

In subjects EC11, EC38 and EC52, most proviral clones encode full-length, intact *vif* and *vpr* open reading frames without known attenuating polymorphisms. Subject EC35 displayed stop codons in a substantial fraction of *vif* (33%) and *vpr* (67%) proviral clones. Subject EC36 exhibited the attenuating polymorphisms Vpr-Q3R + R77Q combined in all proviral clones. Subject EC17 showed stop codons in 20-30% of *vif-vpr* proviral clones, hypermutated sequences in 20% of *vif* proviral clones, and the attenuating polymorphism Vpr-R77Q in all proviral clones. Subject EC19 presented stop codons in 8-17% of *vif-vpr* proviral sequences, hypermutated sequences in 25% of *vif-vpr* proviral clones, and the polymorphisms Vif-R132S+Ins61(EDK) and Vpr-R77Q in all clones analysed. Finally, subject EC42 displayed stop codons in 25-38% of *vif-vpr* proviral sequences, hypermutated sequences in 25% of *vif* proviral clones, and the polymorphisms Vif-T20A+R132S and Vpr-R77Q in most (> 80%) proviral clones.

**MAIN CONCLUSIONS:**

Mutations associated with attenuation of HIV-1 Vif and/or Vpr functions may contribute to the long-term control of viral replication and disease progression in certain ECs.

Elite Controllers (ECs) are a scarce portion of individuals who control human immunodeficiency virus type 1 (HIV-1) replication in the absence of antiretroviral therapy.^1^ Most ECs also control the progression of acquired immunodeficiency syndrome (AIDS) and are thus classified as long-term non-progressors (LTNPs). ECs are a heterogeneous group with multiple mechanisms probably associated with this virologic control scenario.^2^ Several studies show that most ECs were infected by competent and pathogenic viruses,^3^ ^,^ ^4^ ^,^ ^5^ ^,^ ^6^ supporting the relevance of host immune factors in the control of viral replication. Low levels of viremia present in some individuals, however, could be associated with the presence of attenuated HIV-1 strains.^7^ ^-^ ^14^

Accessory genes *vif* and *vpr* are essential in the context of natural infections (*in vivo*) as they encode proteins that regulate wide-ranging aspects of virus replication including controlling antiviral factors present in the innate immune response and increase in viral infectivity, ensuring the viral infection success.^15^ The *vif* gene encodes the virion infectivity factor (Vif) protein that enhances viral replication and counteracts the antiviral effects of apolipoprotein B mRNA editing enzyme (APOBEC), mainly APOBEC3G and APOBEC3F that inhibit viral replication by inducing hypermutations in the HIV-1 genome.^16^ The viral protein R (Vpr) of HIV-1 is a conserved protein encoded by the *vpr* gene^17^ that can induce early activation of non-activated T cells,^18^ facilitating productive HIV-1 infection, and also seems essential for HIV-1 replication in primary monocytes and macrophages.^19^ ^,^ ^20^ ^,^ ^21^ Among the main functions^22^ of Vpr are 1) the import of the HIV-1 pre-integration complex (PIC) into the nucleus;^23^ 2) induction of cell cycle arrest in the G2 phase;^24^ ^,^ ^25^ 3) modulation of T cell apoptosis;^26^ ^,^ ^27^ and 4) transcriptional modulation of viral and host genes.^28^

Some studies have pointed out the importance of insertions, deletions, and premature stop codons in the *vif* and *vpr* genes of some LTNPs.^3^ ^,^ ^7^ ^,^ ^10^ ^,^ ^29^ ^-^ ^32^ Some Vif polymorphisms (I107T and R132S) have been shown to reduce viral fitness *in vitro* and were overrepresented in LTNPs/ECs compared to other HIV-1-infected individuals.^12^ ^,^ ^33^ Other Vif polymorphisms (V13I, V55T, and L81M) were associated with ECs/LTNPs status, but their impact on viral infectivity remains to be determined.^11^ Another study indicates that attenuated anti-APOBEC3G activity of Vif in some ECs resulted from various combinations of minor polymorphisms.^34^ Several Vpr polymorphisms (Q3R, E29G, V31D, S41G, E17S+G43R, E21K+I80T, Q44R+I83V, E58G+A59V, F72L and R77Q/A/H) have also been associated with both LTNPs profile and reduction in *vpr*-induced activities.^8^ ^,^ ^9^ ^,^ ^13^ ^,^ ^35^ ^-^ ^38^ Other Vpr polymorphisms like the simultaneous presence of alanine at position 55 (Ala-55) and threonine (Thr-63) at position 63 would be associated with a low level of plasma viral load and a higher CD4^+^ T lymphocyte counts in chronic HIV-1-infection, but their functional impact on viral infectivity remains to be determined.^14^

To test if attenuation of the function of HIV-1 accessory proteins may explain the long-term control of viral replication in some ECs of our cohort, we performed the single genome amplification of the accessory genes *vif* and *vpr* in samples taken from eight patients with > 10 years of suppressive viral control and no disease progression. Identifying the virologic characteristics present in our cohort of ECs can be important to understanding the mechanisms responsible for the long-term remission profile and provide unique information for treatment strategies of HIV functional cure.

## SUBJECTS AND METHODS

*Study subjects* - In this study, we include eight ECs infected with HIV-1 for at least 10 years that maintained undetectable RNA viral loads in most (> 70%) determinations (n = 5) or in all (100%) determinations (n = 3) throughout follow-up without antiretroviral therapy. Individuals have been followed up at the Instituto Nacional de Infectologia Evandro Chagas (INI) from Rio de Janeiro (Brazil) and provided written informed consent documents approved by the INI Institutional Review Board (Addendum 049/2010) and the Brazilian National Human Research Ethics Committee (CONEP 14430/2011). Patients were followed at least once every 6-12 months to perform infection-monitoring tests such as RNA viral load quantification and CD4^+^ T lymphocyte count. In each visit, PBMC was obtained by Histopaque-1077 (Sigma, USA) density gradient and stored in liquid nitrogen until use. Plasma VL and CD4+ T cells were measured according to the Brazilian Ministry of Health guidelines. Absolute CD4+ T cell counts were obtained using the MultiTest TruCount kit and the MultiSet software on a FACSCalibur flow cytometer (BD Biosciences San Jose, CA). Plasma VL was measured with the Versant HIV-1 3.0 RNA assay (bDNA 3.0, Siemens, Tarrytown, NY, limit of detection: 50 copies/mL) from 2007 to 2013, and the Abbott RealTime HIV-1 assay (Abbott Laboratories, Wiesbaden, Germany, limit of detection: 40 copies/mL) from 2013 to until now.

*Genomic DNA isolation and single genome amplification (SGA) of the vif and vpr genes* - A total of 1 × 10^7^ cryopreserved PBMCs were thawed, and washed, and immediately after, the total genomic DNA was isolated with the addition of the DNAzol^®^ Reagent (Invitrogen, USA) under conditions recommended by the manufacturers. To limit template resampling, SGA was performed using a nested polymerase chain reaction (PCR)-based limiting dilution assay.^39^ To this end, the extracted DNA was diluted until no more than 30% of the reactions were positive after nested PCR, providing a > 70% probability that a single viral template was present in each positive PCR reaction mixture. The amplification of *vif* and *vpr* accessory genes from PBMC-DNA was performed by nested PCR (fragment ~1,000 bp) using AmpliTaq Gold^®^ 360 DNA Polymerase (Applied Biosystems, USA) under conditions recommended by the manufacturers. Supplementary data (Table I) shows the primers used for the two steps of amplification. The final PCR products were purified using the Illustra GFX PCR DNA purification kit (GE Healthcare, USA).

*Sequencing and sequence analysis* - Sequencing reactions were performed using the ABI BigDye Terminator v.3.1 reaction Kit (Applied Biosystems, Foster City, CA) run on an ABI PRISM 3100 automated sequencer (Applied Biosystem). The chromatograms were assembled into contigs using the SeqMan 7.0 software (DNASTAR Inc., Madison, WI) and inspected manually to discard chromatograms of low quality and with double peaks (multiple nucleotides at a single position indicating more than one template per sequencing reaction). The Geneious software v.9.1.8 was used to separate the consensus sequences of the *vif* and *vpr* genes and assemble the multi-alignments (sequences clones for each patient) of both genes. The *vif* and *vpr* sequences were aligned with HIV-1 group M subtypes reference sequences obtained from Los Alamos Database (https://www.hiv.lanl.gov/content/sequence/NEWALIGN /align.html) using ClustalW and then manually edited, yielding a final alignment covering positions 5,041-5,619 of *vif* and 5,559-5,850 of *vpr* relative to the HXB2 reference genome (Genbank accession number: K03455.1). Maximum-likelihood (ML) phylogenetic trees were reconstructed with the PhyML 3.0 program^40^ using the most appropriate nucleotide substitution model (GTR+I+G) selected using program jModeltest v. 3.7,^41^ the SPR branch swapping heuristic tree search algorithm, and the approximate likelihood-ratio test (aLRT)^42^ for branch support. We evaluated the presence of premature stop codons (PSC), frameshift indels, and evidence of APOBEC3G/F mediated hypermutation as determined using Hypermut software.^43^ We also evaluated the frequency of three types of polymorphisms (amino acid substitutions) in HIV-1 Vif and Vpr accessory genes of our cohort of ECs: 1) polymorphisms that have been observed at high frequency in LTNPs respect to control groups and may have a strong functional impact; 2) polymorphisms detected in a few LTNPs/ECs and that have unknown functional impact; and 3) mutations in Vif and Vpr that have an impact on the biological functions of accessory genes, but were not previously associated with LTNPs/ECs [Supplementary data (Table II)].

## Availability of data

The sequences generated in this study were deposited in GenBank^®^ under accession numbers PQ666556-PQ666625.

## RESULTS

*Clinical and epidemiological characteristics* - Our study population of ECs is 88% female (7/8), with a median age of 46 years (IQR: 42-60). Of the eigh individuals, four had their positive HIV-1 diagnosis for just over 10 years, one for more than 20 years, and the remaining three had been living with HIV-1 for more than 25 years (Table I). All ECs in the study were not on antiretroviral therapy and, even during this long period of infection, maintained more than 70% of HIV-1 viral load measurements undetectable and stable CD4^+^ T lymphocyte counts > 500 cells/mm^3^. The HIV-1 viral load and CD4^+^ T cell counts throughout the follow-up of the subjects in this study were presented in detail previously.^44^ Here, in the visit where we accessed the genetic diversity of HIV-1 accessory genes, only two subjects displayed detectable viral loads (61 and 96 viral copies/mL) and the median of CD4^+^ T cells was 1,059 cells/mm^3^ (IQR: 969-1,222) (Table I).

**TABLE I:** Clinical and epidemiological characteristics of Elite Controllers (ECs)

EC	Gender	First HIV-1 positive test (year)	Age (year)	Year of the analysed visit	RNA VL at visit	Undetectable RNA VL in follow-up	CD4^+^ T cells at visit
11	Female	1995	44	2011	< LD	96%	1,590
17	Female	2000	63	2013	96	84%	2,697
19	Male	2006	46	2014	< LD	95%	830
35	Female	2011	38	2018	< LD	100%	977
36	Female	2010	41	2017	< LD	88%	1,077
38	Female	2011	43	2019	< LD	100%	1,041
42	Female	1993	59	2013	61	76%	967
52	Female	1997	48	2019	< LD	100%	1,158

VL: viral load (copies/mL); LD: limit of detection (< 40 copies/mL); CD4^+^T cell (cells/μL).

**Fig. 1:**
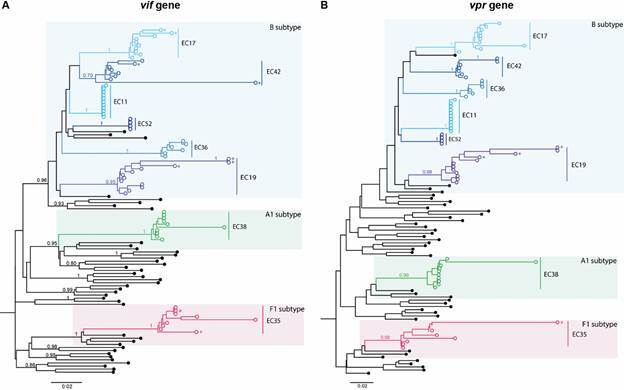
maximum-likelihood (ML) phylogenetic tree of two human immunodeficiency virus type 1 (HIV-1) accessory genes *vif* (A) and *vpr* (B). Sequences obtained from human peripheral blood mononuclear cells (PBMCs) of Elite Controllers (ECs) were combined with HIV-1 subtype reference sequences. Branches were coloured according to subtype assignment. The individual’s identification is displayed on the right side of the clusters. Black circles indicate the reference sequences, and asterisks highlight the sequences with APOBEC3G-mediated G to A hypermutations. The values ≥ 0.80 of approximate likelihood-ratio test (aLRT) support are displayed near the nodes of each cluster. Horizontal branch lengths are proportional to the bar at the bottom, indicating nucleotide substitutions per site.

*Genetic characterisation of accessory genes* - We obtained proviral sequences of *vif* and *vpr* genes from all individuals. The sequences obtained for each accessory gene were aligned with reference sequences and subject to ML phylogenetic analysis. The phylogenetic tree of each gene showed that sequences from each individual cluster together with high support (aLRT), indicating the monophyletic origin of viruses infecting each individual (Fig. 1). The only exception was the cluster of *vif* sequences of subject EC42 that display a lower support (aLRT = 0.70) probably due to the presence of a basal hypermutated divergent sequence. All individuals showed congruence in the subtype classification of both *vif* and *vpr*: 75% (6/8) were classified as subtype B, 12.5% as subtype F1 (1/8), and 12.5% as subtype A1 (1/8) (Fig. 1).

The total number of proviral clones per individual ranged from 4 to 12 (Table II). None of the proviral clones obtained for *vif* and *vpr* in our ECs cohort showed frameshift indels. Three individuals (EC11, EC36, and EC52) presented functional sequences at all proviral clones analysed, without PSC, indels or APOBEC3G/F mediated hypermutations. Subject EC38 displayed proviral clones with PSC in only a minor fraction (10%) of *vif* and *vpr* sequences analysed. Individual EC19 displayed PSC in 17% of *vif* and 8% of *vpr* proviral sequences, hypermutated sequences in 25% of *vif*/*vpr* proviral clones, and an inframe insertion of three amino acids (EDK) at position 61 [Ins61(EDK)] in all *vif* proviral sequences. Individuals EC17, EC35 and EC42 displayed PSC in 30-38% of *vif* and 20-67% of *vpr* proviral clones and hypermutated sequences in 20-25% of *vif* and < 11% of *vpr* proviral clones.

**TABLE II:** Stop codons, indels, and APOBEC3G/F mediated hypermutation in the *vif* and *vpr* proviral sequences

EC	No of clones	*vif* gene					*vpr* gene				
		wt	ins	del	PSC	Hyp	wt	ins	del	PSC	Hyp
11	11	11	-	-	-	-	11	-	-	-	-
17	10	7	2	-	3	2	8	-	-	2	-
19	12	10	12	-	2	3	11	-	-	1	3
35	9	6	-	1	3	2	3	-	-	6	1
36	6	6	-	-	-	-	6	-	-	-	-
38	10	9	-	-	1	-	9	-	-	1	-
42	8*	5	-	-	3	2	5	-	-	2	-
52	4	4	-	-	-	-	4	-	-	-	-

PSC: premature stop codon; wt: wild type; ins: insertion; del: deletion; Hyp: hypermutated. ^*^One sequence was obtained partially and only for the *vif* gene for this patient. Null values are shown as a hyphen in the table.

**Fig. 2:**
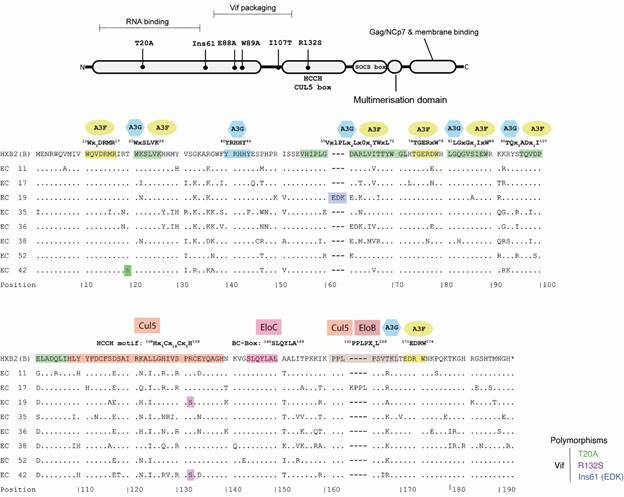
human immunodeficiency virus type 1 (HIV-1) Vif protein. (A) Schematic representation of the functional domains of the protein and the locations of mutations that attenuate its function. (B) Alignment of deduced consensus amino acid sequences for the Vif protein of Elite Controllers (ECs). Amino acids with a frequency greater than 60% among proviral sequences from each individual are represented in the consensus. Dots indicate amino acids identical to those in the HXB2 reference sequence, which is shown on the top line. Major functional domains are represented at the top of the sequences within the boxes and highlighted in the reference sequence. The colours highlighted in the consensus sequences indicate polymorphisms described as enriched in long-term non-progressors (LTNPs) [R132S and Ins61 (EDK)] or not (T20A), as shown in the legend.

Some Vif polymorphisms described in previous studies as being more enriched in ECs/LTNPs compared with non-controllers and/or being associated with the reduction of accessory protein biological activities were identified in our cohort (Fig. 2). Of the Vif polymorphisms described to be enriched in LTNPs that also cause a functional impact on the protein (I107T, R132S, and Ins61), the R132S was observed in our cohort in all clones of individual EC19, and the majority (80%) of proviral clones from subject EC42. The EC19 also showed an insertion of three amino acids (EDK) between codons 60 and 61 [Ins61(EDK)]. Of the polymorphisms with functional impact but without prior association with LTNPs/ECs (T20A, E88A+W89A, C114A/S, F115A, R132A, C133A/S), we detected the T20A in all sequences of individual EC42.

**Fig. 3:**
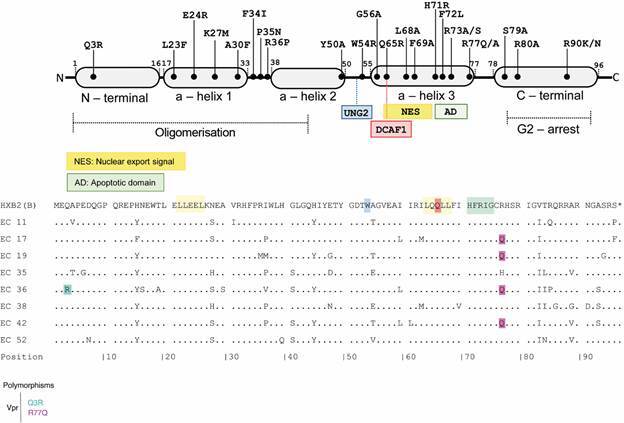
human immunodeficiency virus type 1 (HIV-1) Vpr protein. A) Schematic representation of functional domains of the protein and locations of mutations that attenuate protein function. B) Alignment of deduced consensus amino acid sequences for the Vpr protein of Elite Controllers (ECs). Amino acids with frequency greater than 60% among proviral sequences from each individual are represented in the consensus. The dots indicate the amino acids identical with those of the HXB2 reference sequence, which is shown on the top line. Major functional domains are represented at the top of the sequences within the boxes and highlighted in the reference sequence. The colors highlighted in the consensus sequences indicate polymorphisms described as enriched in long-term non-progressors (LTNPs) (Q3R and R77Q), as shown in the legend.

Some relevant Vpr polymorphisms were also identified in our cohort (Fig. 3). Of the Vpr polymorphisms that have already been observed in LTNPs/ECs individuals and that have an impact on the functionality of the protein (Q3R, Q65R, F72L and R77Q), the R77Q was detected in all proviral clones of four ECs (EC17, EC19, EC36 and EC42) and, we further detected the polymorphism R77H in all proviral clones of subject EC35. The Q3R+R77Q polymorphisms combined were detected in all proviral of individual EC36 and a single proviral clone of subject EC42. Of the polymorphisms related to the LTNPs/ECs phenotype but without changes in Vpr functionality (T19A and R90N), individual EC36 presented the T19A polymorphism in all clones. Of the polymorphisms that impact Vpr functionality but without prior association with LTNPs/ECs individuals, we found the R90K in one clone of individuals EC19 and EC35.

## DISCUSSION

In this study, we evaluated the possibility of attenuation of HIV-1 replicative capacity in eight ECs who control viral load for long periods without disease progression through analysis of proviral quasispecies of accessory genes *vif* and *vpr*. Accessory genes are essential in the context of natural infections (*in vivo*) as they encode proteins that act both to control the response of some antiviral factors present in the innate immune response and to assist in changes in the cellular machinery that favor an increase in viral infectivity, ensuring the viral infection success.^15^ Overall, we detected a high frequency of full-length *vif* and *vpr* sequences without gross deletions in the viral quasispecies of our cohort of ECs, which is in line with a previous study that revealed that large deletions in accessory genes other than *nef* are very infrequent in ECs.^45^

Several proviral clones comprising stop codons, small indels or key polymorphisms were detected in our cohort, although with variable frequency across ECs. Three individuals (EC11, EC38 and EC52) presented sequences without evidence of stop codons or indels at most (> 90%) proviral clones analysed. These individuals further displayed proviral clones without polymorphisms in accessory genes previously associated with altered protein function, supporting infection by HIV-1 variants harboring fully functional Vif and Vpr proteins. Subject EC35 displayed stop codons in a substantial fraction of *vif* (33%) and *vpr* (67%) proviral clones; but no attenuating mutations in accessory genes. Subject EC36 displayed some key polymorphisms in Vpr and subject EC19 displayed some polymorphisms in both Vif and Vpr. Subject EC17 displayed a key polymorphism in Vpr and stop codons in a substantial fraction of *vif* (30%) and *vpr* (20%) proviral clones. Finally, subject EC42 displayed some key polymorphisms in both Vif and Vpr as well as stop codons in a significant fraction (25-38%) of *vif* and *vpr* proviral sequences.

A large proportion of proviral sequences of individual EC42 (80%) displayed two key Vif polymorphisms, T20A and R132S, that combined may have a synergistic effect in reducing Vif activity. A recent study demonstrates that Vif is stabilised through AKT-mediated phosphorylation at threonine 20, which potentiates HIV-1 infectivity, and that Vif mutant T20A is less stable than Vif wild type.^46^ Meanwhile, mutation R132S takes place in the motif responsible for the interaction of Vif with the cellular Cul5-based ubiquitin ligase E3 that targets APOBEC3G/3F for proteasomal destruction, and *in vitro* studies have shown that this substitution reduced the replicative capacity of HIV-1 in activated PBMCs.^33^ ^,^ ^47^ Of note, the Vif mutant R132S is detected at a higher frequency in LTNPs with low viral load than in LTNPs with high viral loads or typical progressors.^33^ These findings support a putative association between Vif polymorphisms T20A+R132S and the low HIV-1 RNA viral load *in vivo* in subject EC42.

The Vif R132S polymorphism was also detected in all proviral clones of individual EC19, together with a three-amino-acid insertion (EDK) between positions 60-61 of Vif [Ins61EDK)]. Notably, a previous study revealed a two-amino-acid insertion (DS) in that same position of Vif in viruses recovered from a LTNPs mother-child pair with consistently low plasma RNA viral loads.^30^ That study also showed that the HIV-1 isolated from the LTNP child replicated poorly in PBMCs and further proved that the two-amino-acid insertion in Vif resulted in abnormally low concentration of full-length Vif and was the primary determinant of the poor viral replication capacity. We thus speculate that the combined presence of the Vif polymorphisms R132S+Ins61(EDK) may be a also relevant determinant of the elite control phenotype of patient EC19.

The presence of Vif R132S mutation in most proviral clones of subjects EC19 and EC42 coincides with detection of stop codons and APOBEC3G/F mediated hypermutations in a significant fraction (17-38%) of *vif* proviral sequences, which may have resulted from a lower anti-APOBEC3G/3F Vif activity associated with the attenuating mutations. However, we found no direct association between presence of attenuating mutations in Vif and proportion of stop codons and/or APOBEC3G/F mediated hypermutations in HIV-1 sequences of ECs. Subjects EC17 and EC35 displayed stop codons and/or hypermutations in a substantial fraction of *vif* (20-33%) and *vpr* (20-67%) proviral clones, but no evidence of attenuating mutations in Vif. One hypothesis is that the relative high proportion of *vif* and *vpr* proviral clones with stop codons and/or hypermutations detected in some ECs may be related to the inability of immune system to eliminate cells harboring defective proviruses rather than to attenuated anti-APOBEC3G activity of Vif.^48^ ^,^ ^49^

The Vpr R77Q mutation was the most frequent polymorphism, being observed in all proviral clones of 50% of individuals here analysed (EC17, EC19, EC36 and EC42). The Vpr R77Q polymorphism result in less T cell apoptosis, despite similar levels of viral replication, and this mutation was detected in 30-45% of progressors as compared with 75-90% of LTNPs.^8^ ^,^ ^9^ One remarkable finding was the presence of the Vpr R77H mutation in all proviral clones of subject EC35. A previous study showed that the Vpr R77H polymorphism was more common in individuals infected with HIV-1 subtypes K and F,^37^ as is the case of EC35 that was infected by subtype F1, supporting that this was a subtype-associated polymorphism. It is also interesting to note that all proviral sequences of individual EC36 displayed the Vpr Q3R polymorphism in addition to R77Q. The Vpr Q3R polymorphism was previously detected in a viremic LTNPs and similar to mutation R77Q it was shown to markedly impair the cytopathic and proapoptotic activity of Vpr without affecting viral replication efficiency.^35^ Thus, these findings support a putative association between Vpr polymorphisms and low virulence of infecting viruses in subjects EC17, EC19, EC36 and EC42.

It is interesting to note that polymorphisms in Vif (T20A and R132S) and/or Vpr (Q3R and R77Q) were detected in four out of five ECs with occasional blips and none out of three ECs with persistent undetectable viremia of our cohort.^44^ This suggests that attenuating Vif and Vpr polymorphisms may be more frequent in ECs with higher residual viral replication. This could be particularly important in subjects EC19 and EC42 that displayed attenuating mutations in both Vif and Vpr proteins simultaneously. All clones from subject EC19 displayed the polymorphisms Vif-R132S+Ins61(EDK)+Vpr-R77Q, while nearly all clones from subject EC42 displayed the polymorphisms Vif-T20A+R132S+Vpr-R77Q. The combined attenuation of both Vif and Vpr proteins may have not only additive but also synergistic effects on HIV-1 pathogenesis. Vif and Vpr independently drive G2/M cell cycle arrest and apoptosis of CD4+ T-cells *in vitro*, and are thus necessary for the HIV-1-induced T-cell cytopathicity.^50^ ^,^ ^51^ Moreover, Vif and Vpr can independently contribute to the attenuation of the innate antiviral response by inducing degradation of IRF-3 in T-cells and inhibition of TBK1 in human dendritic cells and macrophages.^52^ ^,^ ^53^

Our study has some limitations. First, we evaluate a limited number of individuals. With recommendations favorable to the increasingly earlier initiation of antiretroviral therapy, identifying individuals with a natural control profile is increasingly scarce, making it very difficult to recruit new individuals in the cohort. In addition, a minimum of five years of follow-up is required to confirm “stability” of natural control and achieving long-term follow-up remains a significant challenge. Second, we assessed only a limited number of proviral clones, so we cannot rule out the possibility that a small proportion of *vif* and *vpr* sequences without attenuating mutations may still persist within the viral quasispecies. Third, our study does not evaluate the functionality of the accessory proteins recovered from the subjects. Moreover, a substantial fraction of non-ECs also display the mutations Vif-T20A (18%), Vif-R132S (38-57%), Vif-Ins61(EDK) (2%), and Vpr-R77Q (29-42%) in consensus sequences, suggesting that these polymorphisms alone are insufficient to account for the elite control status.^8^ ^,^ ^9^ ^,^ ^13^ ^,^ ^34^ ^,^ ^54^ However, those previous studies did not demonstrate whether these Vif-Vpr polymorphisms were dominant within the viral quasispecies of non-ECs, as we have shown here for ECs.

In summary, our study reveals a high frequency of full-length *vif* and *vpr* sequences in the viral quasispecies analysed, excluding the possibility that gross genetic defectecs within those accessory genes contribute to the long-term EC status of the individuals included in our cohort. However, key amino acid changes within Vif [T20A, R132S, and Ins61(EDK)] and/or Vpr (Q3R and R77Q) proteins that attenuate viral replication were detected in most proviral clones of several long-term ECs here analysed, particularly in those with sporadic blips. Moreover, we detected two ECs with proviral quasispecies mostly composed by sequences harboring attenuation mutations in both Vif and Vpr proteins simultaneously. Although these polymorphisms are also detected in a minor fraction of individuals with progressive disease, indicating they do not fully-explained the status of elite control, the presence of HIV-1 strains with Vif and/or Vpr attenuating mutations, combined with protective host factors, may contribute to the long-term control of viral replication and disease progression in some ECs.
