# Supplementary material for: Identification of polymorphisms associated with attenuation of Vif and Vpr in HIV-1 Elite Controllers
Source: Mem Inst Oswaldo Cruz. 2025 Jun 27;120:e240274. doi: 10.1590/0074-02760240274 (PMC12208676; doi:10.1590/0074-02760240274)
Supplement: Supplementary file 1 [file 1678-8060-mioc-120-e240274-s.pdf]

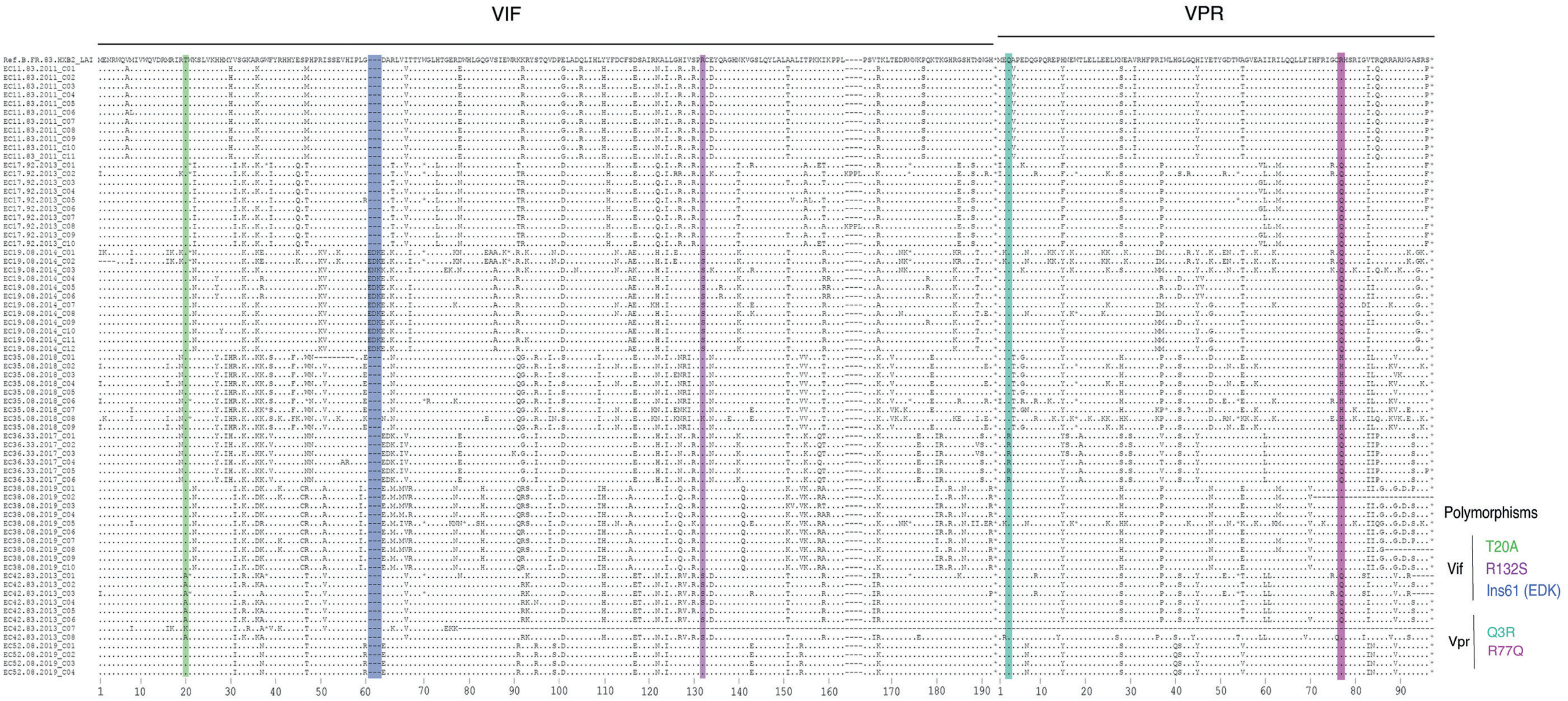

Alignment of all amino acid clones sequences generated for the Vif and Vpr proteins of ECs. Dots indicate amino acids identical to those in the HXB2 reference sequence, which is shown on the top line. Asterisks indicate regions with premature and final stop codons for each protein. Gaps were identified with a dash (-). The highlighted colors in the amino acid sequences indicate polymorphisms described as enriched in LTNPs [Vif: R132S and Ins61 (EDK); Vpr: Q3R and R77Q] or not (Vif: T20A), as shown in the legend.

TABLE I

Amplification strategy of *vif* and *vpr* HIV-1 accessory genes

1° PCR amplification	Fragment Size*	2° PCR amplification	Fragment Size*
SCCOS(Fw): 5'TACAGTGCAGGGGAAAGAATARTAGACATAATA 3	1,170pb	SCCNS (Fw): 5'CAAAATTTCCGGGTTTATTACAGGGACA 3'	1,087pb
ED3AS (Rv): 5'CCTGCCATAGGARATGCCTAA 3'		ED3AS (Rv): 5'CCTGCCATAGGARATGCCTAA 3'	

PCR: polymerase chain reaction; Fw: forward; Rv: reverse; *Fragment size in relation the HXB2.

cc BY

TABLE II

Summary of HIV-1 genetic mutations in *vif* and *vpr* accessory genes

Protein	Polymorphism	Enriched in ECs/LTNPs	Functional impact	Reference
Vif	T20A	Not determined	Reduced Vif stability and HIV-1 replication in PBMCs	(46)
	E88A+W89A		Reduced Vif expression and HIV-1 replication in H9 cells	(55)
	C114A/S		ΔVif phenotype	(47,56)
	F115A			
	R132A			
	C133A/S			
	I107T	Yes	Reduced HIV-1 replication in PBMCs	(12)
	R132S			(33)
	Ins61(DS)			(30)
	V13I		Not determined	(11)
	V55T			
	L81M			
	K22E		Reduced HIV-1 infectivity	(57)
	S32P			
	Y40H			
	E45G			
	F115S			
	G138R			
	L150P			
Vpr	Q3R	Yes	Reduce the cytopathicity	(35)
	Q65R		Failed to induce G2-arrest and cell death	(58)
	F72L		Reduce incorporation of Vpr into the forming virions	(38)
	R77Q		Reduce the cytopathicity	(8,9,13,37)
	R90N		Not determined	(13)
	L23F	Not determined	1) Nucleoporin binding mutant, diffuse nucleocytoplasmic distribution, 2) unable to cause G2/M arrest	(59)
	E24R, R36P		Selectively disrupts Vpr binding to helicase-like transcription factor (HLTF) without disturbing the UNG2 degradation	(60,61)
	K27M		1) Nucleoporin binding mutant, diffuse nucleocytoplasmic distribution, 2) no G2 arrest, 3) no apoptosis induction, 4) no accumulation at the nuclear envelope	(62)
	A30F		Unable to bind gag and package into particles	(63)
	F34I		1) Decreased Vpr incorporation into virions compared to WT Vpr, 2) Unable to bind the nuclear envelope but causes G2/M arrest	(23,64)
	P35N		Unable to interact with cyclophilin A	(65)
	Y50A		G2/M arrest defective mutant	(13)
	W54R		Defective for UNG2 loading onto CRL4 but retains binding to DCAF	(62)
	G56A		Defective for HLTF degradation, ability to arrest cell cycle maintained	(66)
	L68A		Nuclear export mutant	(67)
	F69A		DCAF binding mutant, but associates with Exo1 like wild type Vpr	(68)
	H71R		1) DCAF binding mutant, unable to induce G2/M cell cycle arrest, 2) Decreased Vpr incorporation into virions	(25,64,69)
	R73A/S		Reduce the cytopathicity	(37,70)
	R90K		Relieves IL-12 suppression	(71)
	R85QRR		Impaired nuclear targeting by the virus	(72)

REFERENCES

55. Fujita M, Sakurai A, Yoshida A, Miyaura M, Koyama AH, Sakai K, et al. Amino acid residues 88 and 89 in the central hydrophilic region of human immunodeficiency virus type 1 Vif are critical for viral infectivity by enhancing the steady-state expression of Vif. J Virol. 2003; 77(2): 1626-32.
56. Sakai K, Horiuchi M, Iida S, Fukumori T, Akari H, Adachi A. Mutational analysis of human immunodeficiency virus type 1 vif gene. Virus Genes. 1999; 18(2): 179-81.
57. Simon V, Zennou V, Murray D, Huang Y, Ho DD, Bieniasz PD. Natural variation in Vif: differential impact on APOBEC3G/3F and a potential role in HIV-1 diversification. PLoS Pathog. 2005; 1(1): e6.
58. Jacquot G, Le Rouzic E, Maidou-Peindara P, Maizy M, Lefrère JJ, Daneluzzi V, et al. Characterization of the molecular determinants of primary HIV-1 Vpr proteins: impact of the Q65R and R77Q substitutions on Vpr functions. PLoS ONE. 2009; 4(10): e7514.
59. Jacquot G, Le Rouzic E, David A, Mazzolini J, Bouchet J, Bouaziz S, et al. Localization of HIV-1 Vpr to the nuclear envelope: impact on Vpr functions and virus replication in macrophages. Retrovirology. 2007; 4(1): 84.
60. Hrecka K, Hao C, Shun MC, Kaur S, Swanson SK, Florens L, et al. HIV-1 and HIV-2 exhibit divergent interactions with HLTF and UNG2 DNA repair proteins. Proc Natl Acad Sci. 2016; 113(27). Available from: https://pnas.org/doi/full/10.1073/pnas.1605023113.
61. Zhou X, DeLucia M, Hao C, Hrecka K, Monnie C, Skowronski J, et al. HIV-1 Vpr protein directly loads helicase-like transcription factor (HLTF) onto the CRL4-DCAF1 E3 ubiquitin ligase. J Biol Chem. 2017; 292(51): 21117-27.
62. Maudet C, Bertrand M, Le Rouzic E, Lahouassa H, Ayinde D, Nisole S, et al. Molecular insight into how HIV-1 Vpr protein impairs cell growth through two genetically distinct pathways. J Biol Chem. 2011; 286(27): 23742-52.
63. Bachand F, Yao XJ, Hrimech M, Rougeau N, Cohen ÉA. Incorporation of Vpr into human immunodeficiency virus type 1 requires a direct interaction with the p6 domain of the p55 Gag precursor. J Biol Chem. 1999; 274(13): 9083-91.
64. Miller CM, Akiyama H, Agosto LM, Emery A, Ettinger CR, Swanstrom RI, et al. Virion-associated Vpr alleviates a postintegration block to HIV-1 infection of dendritic cells. J Virol. 2017; 91(13): e00051-17.
65. Zander K, Sherman MP, Tessmer U, Bruns K, Wray V, Prechtel AT, et al. Cyclophilin A interacts with HIV-1 Vpr and is required for its functional expression. J Biol Chem. 2003; 278(44): 43202-13.
66. Lahouassa H, Blondot ML, Chauveau L, Chougui G, Morel M, Leduc M, et al. HIV-1 Vpr degrades the HLTF DNA translocase in T cells and macrophages. Proc Natl Acad Sci. 2016; 113(19): 5311-6.
67. Jenkins Y, Sanchez PV, Meyer BE, Malim MH. Nuclear export of human immunodeficiency virus type 1 Vpr is not required for virion packaging. J Virol. 2001; 75(17): 8348-52.
68. Wu Y, Zhou X, Barnes CO, DeLucia M, Cohen AE, Gronenborn AM, et al. The DDB1-DCAF1-Vpr-UNG2 crystal structure reveals how HIV-1 Vpr steers human UNG2 toward destruction. Nat Struct Mol Biol. 2016; 23(10): 933-40.
69. Schröfelbauer B, Yu Q, Zeitlin SG, Landau NR. Human immunodeficiency virus type 1 Vpr induces the degradation of the UNG and SMUG uracil-DNA glycosylases. J Virol. 2005; 79(17): 10978-87.
70. Jacotot E, Ravagnan L, Loeffler M, Ferri KF, Vieira HLA, Zamzami N, et al. The HIV-1 Viral protein R induces apoptosis via a direct effect on the mitochondrial permeability transition pore. J Exp Med. 2000; 191(1): 33-46.
71. Tcherepanova I, Starr A, Lackford B, Adams MD, Routy JP, Boulassel MR, et al. The immunosuppressive properties of the HIV Vpr protein are linked to a single highly conserved residue, R90. PLoS ONE. 2009; 4(6): e5853.
72. Zhou Y, Lu Y, Ratner L. Arginine residues in the C-terminus of HIV-1 Vpr are important for nuclear localization and cell cycle arrest. Virology. 1998; 242(2): 414-24.
